# Upper-Extremity Injuries in a Level 1 Trauma Center Following Border Wall Height Increase

**DOI:** 10.1016/j.jhsg.2024.07.002

**Published:** 2024-08-30

**Authors:** Emma Williams, Vivian Hu, Cooper Haaland, Meera Reghunathan, Laura N. Haines, Jay J. Doucet, Todd W. Costantini, Katharine M. Hinchcliff

**Affiliations:** ∗ School of Medicine, University of California, San Diego, San Diego, CA, USA; † Division of Plastic Surgery, Department of Surgery, University of California, San Diego, San Diego, CA, USA; ‡ Division of Trauma, Surgical Critical Care, Burns and Acute Care Surgery, Department of Surgery, University of California, San Diego, San Diego, CA, USA

**Keywords:** Border, Fall, Fracture, Trauma

## Abstract

**Purpose:**

From 2018–2019, the height of over 400 miles of southern border wall was raised to 30 feet. Our aim was to evaluate the impact of the increase in border wall height on upper-extremity injuries sustained via barrier fall.

**Methods:**

A retrospective review of patients admitted with upper-extremity injuries sustained via border wall fall between January 2015 and December 2022 at a Level 1 trauma center serving the United States-Mexico border. Patients admitted between 2015–2018 were included in the preincrease group, and those admitted between 2019–2022 were included in the postincrease group. Demographic data, injury severity metrics, fracture characteristics, operative treatments, hospital charges, and lengths of stay were compared.

**Results:**

In total, 110 patients were identified, with 16 preincrease and 94 postincrease. Following the barrier height increase, patients had higher injury severity scores. Radial fractures were most common pre- and postincrease and accounted for nearly one-third of all fractures. Postincrease upper-extremity trauma patients required more operative events (2.15 ± 2.10 vs 1.44 ± 0.73 preincrease). The average cost for each patient’s hospital stay also quadrupled after the increase in wall height ($397,632 ± $1,057,574 vs $98,978 ± $84,169 preincrease).

**Conclusions:**

The increase in overall injury severity and costly inpatient treatment of upper-extremity injuries among patients who fell from the border following construction has placed additional stress on an already strained health care system.

**Level of Evidence:**

Differential Diagnosis/Symptom Prevalence Study, IV.

There are frequent border crossings at the United States border. In 2022, a single port of entry reported over 15 million passenger vehicle and 6 million pedestrian authorized crossings, which does not capture potential unauthorized crossings occurring through official ports of entry.^1^^,^^2^ Outside recognized ports, unauthorized border crossings also occur along the remaining more than 100 miles of southern border. In early 2017, Executive Order 13767 was signed with the aim of deterring unauthorized crossings by fortifying the physical barrier along the border.^3^ This fortification was completed over the course of 2018–2019, with the majority of work performed on existing structures. Although only 50 miles of new barrier were added, the height of over 400 miles of existing 6- to 17-foot high barrier was raised to 30 feet, effectively doubling the height to the equivalent of a two-to-three story building.^4^

Despite the rise in barrier height, unauthorized border crossings have only increased in frequency. In the 2022 fiscal year, US Customs and Border Protection reported over 240,000 migrant encounters in two border sectors, defined as apprehension of a noncitizen who is determined to be inadmissible. More than 80% of these were single adults.^5^ In comparison, a total of just under 45,000 apprehensions, defined as the arrest of a potentially removable noncitizen by the Department of Homeland Security, were reported in 2017.^6^ The majority (60%) of apprehended migrants have Mexican citizenship, though countries across the globe are represented.^5^

Migrants attempting to cross the border through unauthorized ports of entry face serious danger. The most recently available US Customs and Border Protection data report 77 border-related deaths in two border sectors in the 2021 fiscal year, up from six deaths reported in 2017.^7^ Countless more are nonfatally injured, a subset of which are transported to one of two close by hospitals for treatment. These injuries can lead to notable morbidity and health care costs, the magnitude of which has only grown since the increase in barrier height.^8^, ^9^, ^10^, ^11^ Recent work out of a Level 1 trauma center by the border has shown a 5-fold increase in patients admitted with border fall injuries since the completion of barrier construction.^4^ Additional work focusing on spine and lower-extremity injuries has demonstrated increased costs and resources required to treat border fall patients.^12^^,^^13^

Upper-extremity injuries can be a particularly devastating consequence of falls from height.^14^, ^15^, ^16^, ^17^ These injuries can have profound psychological sequelae, and come at great economic cost to the patient and the payor because of the direct costs of resource-intensive treatment and rehabilitation as well as the indirect costs related to lost wages and productivity.^18^, ^19^, ^20^ In this study, we aimed to evaluate the impact of wall height on upper-extremity injuries sustained via barrier fall. We hypothesized the increase in height of the border wall would correlate with an increase in number and severity of upper-extremity injuries and an increase in treatment-related costs.

## Materials and Methods

A retrospective review of patients admitted with upper-extremity injuries sustained via border wall fall between January 2015 and December 2022 at a Level 1 trauma center serving the border region was performed. The trauma registry was used to identify patients admitted to the trauma service with upper-extremity injury during this study period. Patients whose only upper-extremity injury was an abrasion or laceration not requiring repair were excluded. A scapula fracture alone was not considered an upper-extremity injury; however, scapula fractures were recorded for patients included in the analysis. Patients admitted before the height of the wall increased, between 2015 and 2018, were included in the preincrease group. The postincrease group included patients admitted between 2019 and 2022, after the increase in wall height.

Demographic data and injury severity metrics, including the Injury Severity Score (ISS) and upper-extremity Abbreviated Injury Scale (AIS-90), were collected. The presence of fracture outside of the upper-extremity was gathered through the trauma registry. Specific upper-extremity injuries, including fractures, traumatic arthrotomies, dislocations, and lacerations, were recorded. Fractures of the scapula, upper and lower arm, wrist, and hand were identified and characterized based on radiology reports. Presence of upper-extremity traumatic arthrotomy or dislocation was similarly gathered through radiology reports and operative notes during chart review. Only lacerations requiring repair were included in this analysis and were characterized as superficial if they involved only skin and subcutaneous tissues or deep if they involved deeper structures, including muscle, or neurovascular structures. The number and duration of bone and soft tissue operative interventions were recorded. When patients with polytrauma were undergoing multiple interventions during a single trip to the operating room, the duration of only upper-extremity interventions were included. Specific operative interventions, including internal fixation of fractures, grafting for soft tissue injury, or microsurgical procedure for reconstruction, as well as utilization of negative pressure (wound vac) therapy were recorded. Additional data collected included hospital charges and hospital and intensive care unit (ICU) lengths of stay (LOS). Hospital charges were adjusted for inflation and are reported in 2015 US Dollars.

Statistical analyses was performed using the Welch *t* test for continuous variables and Chi-squared or Fisher’s exact test for categorical variables as appropriate. All tests were two-sided, and the significance level was set to α = 0.05.

This study was approved by the institutional review board.

## Results

**Figure 1:**
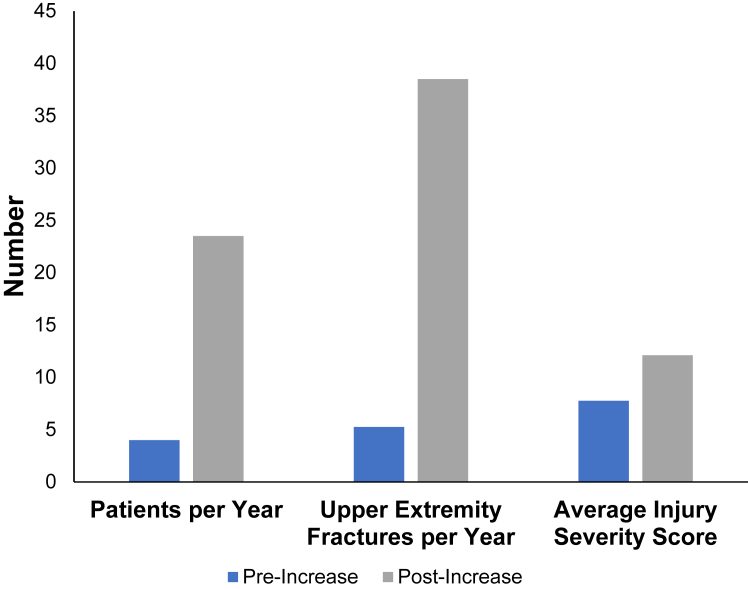
Pre- versus postincrease traumatic upper-extremity injuries sustained via border wall fall. The number of patients with upper-extremity border fall injury, number of upper-extremity fractures because of border fall per year, and average injury severity score of presenting patients all increased following increase in barrier height.

There were a total of 110 patients identified, 16 patients before the border wall increase and 94 patients after the border wall increase, representing greater than 5-fold increase in admissions for patients with upper-extremity injuries after a border wall fall (Fig. 1). Both groups were similar in age, sex, race, and ethnicity (Table 1). Following the increase in border wall height, more patients with upper-extremity trauma secondary to border falls presented primarily to this Level 1 trauma center as opposed to being transferred in from outlying facilities (69% transfers in preincrease, 27% transfers in postincrease, *P* = 0.003).

**Table 1:** Demographics∗

	2015–2018 (n = 16) Avg ± SD^a^ or num (%)	2019–2022 (n = 94) Avg ± SD or num (%)	*P* Value
Age (y)	34.11 ± 12.4	32.3 ± 10.5	0.590
Sex			
M	11 (69%)	71 (76%)	0.548
F	5 (31%)	23 (24%)	
Race			
Native Hawaiian	0 (0%)	2 (2%)	0.640
Other	16 (100%)	89 (95%)	
White	0 (0%)	3 (3%)	
Ethnicity			
Hispanic	15 (94%)	87 (93%)	1.000
Non-Hispanic	1 (6%)	7 (7%)	
Transfer in?			
Yes	11 (69%)	25 (27%)	0.003
No	5 (31%)	69 (73%)	

SD, standard deviation.

∗ This demonstrates the demographics of the patient cohort of border wall injuries with upper-extremity injuries both before and after increase in the border wall height. A *P* value less than 0.05 is considered statistically significant.

### Injury severity

**Figure 2:**
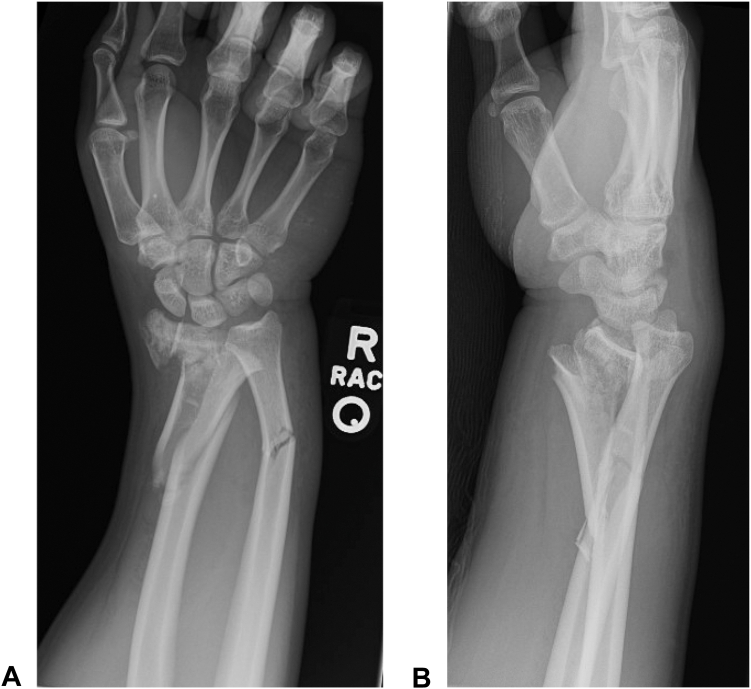
Example of wrist and forearm injury sustained from fall from border wall. **A** Anterior-posterior and **B** lateral x-rays demonstrate an example of an upper-extremity injury with distal fracture of both the radius and the ulna that can be sustained from a fall from significant height.

Following border wall construction, patients had a higher ISS (12.11 ± 7.82 vs 7.75 ± 5.56 preincrease, *P* = 0.012). Patients were more likely to have a concomitant fracture outside of the upper extremities after the border wall height increase (87% vs 50% preincrease, *P* = 0.002) and have a greater number of these other (nonupper extremity) fractures (3.76 ± 2.62 vs 1.81 ± 2.56 preincrease, *P* = 0.011, Table 2). Figure 2 shows the radiographic images for a border wall injury that resulted from a significant fall from height.

**Table 2:** Injury Severity and Characteristics∗

	2015–2018 (n = 16) Avg ± SD or num (percent)	2019–2022 (n = 94) Avg ± SD or num (percent)	*P* Value
ISS	7.75 ± 5.56	12.11 ± 7.82	0.012
AIS Score for upper extremity	1.81 ± 0.40	2.06 ± 1.14	0.110
Upper arm injury	5 (31%)	24 (26%)	0.759
Forearm injury	8 (50%)	68 (72%)	0.086
Wrist/hand injury	8 (50%)	60 (64%)	0.404
Superficial laceration	6 (38%)	33 (35%)	1.000
Deep laceration	5 (31%)	11 (12%)	0.056
Arthrotomy	2 (13%)	8 (9%)	0.637
Dislocation	3 (19%)	17 (18%)	1.000

Fracture characteristics			
No. of UE fractures per patient	1.31 ± 1.25	1.64 ± 1.63	0.367
No. of UE fractures, total	21	154	
Fracture, by bone			
Scapula	0 (0%)	3 (2%)	
Humerus	4 (19%)	18 (12%)	
Radius	7 (33%)	47 (31%)	
Ulna	4 (19%)	43 (28%)	
Any carpal	3 (14%)	26 (17%)	
Any metacarpal	0 (0%)	9 (6%)	
Any phalange	3 (14%)	8 (5%)	
Open fracture	4 (25%)	12 (13%)	0.246
Displaced fracture	10 (63%)	59 (63%)	1.000
Comminuted fracture	6 (38%)	48 (51%)	0.419
Other fractures besides UE	8 (50%)	82 (87%)	0.002
No. of non-UE fractures	1.81 ± 2.56	3.76 ± 2.62	0.011

SD, standard deviation; AIS, abbreviated injury scale; ISS, injury severity score; UE, upper extremity.

∗ This describes details of upper-extremity injuries sustained before and after border wall height increase. Superficial lacerations were defined as lacerations involving only skin and subcutaneous tissues requiring repair. Deep lacerations were defined as lacerations involving deeper structures, including muscle and/or neurovascular structures, that required repair. A *P* value less than 0.05 is considered statistically significant.

Patients had similar AIS scores for upper extremity and did not require significantly increased reconstructive care (eg, negative pressure wound therapy, grafting, and microsurgical intervention) postincrease. Rates of arthrotomy, dislocation, and superficial and deep lacerations were similar between the two groups. Although the total number of upper-extremity fractures increased following border construction, the average number of fractures per patient was not significantly different between groups. Radius fractures were the most common both pre- and postincrease and accounted for nearly one-third of all upper-extremity fractures. Scapula fractures were not seen in patients with upper-extremity injury preincrease but accounted for 2% of fractures postincrease. Fracture characteristics (open, displaced, and comminuted) were similar between groups (Table 2), as were treatments, including rates of debridement, closed reduction, and internal fixation (Table 3).

**Table 3:** Treatment of Upper-extremity Injuries∗

	2015–2018 (n = 16) num (percent)	2019–2022 (n = 94) num (percent)	*P* Value
Closed reduction	11 (69%)	60 (64%)	0.784
Percutaneous pinning	1 (6%)	2 (2%)	0.379
Internal fixation	5 (31%)	37 (39%)	0.591
Debridement	4 (25%)	15 (16%)	0.472
Wound vac	0 (0%)	1 (1%)	1.000
Skin graft	0 (0%)	1 (1%)	1.000
Local flap	0 (0%)	0 (0%)	N/A
Free flap	0 (0%)	0 (0%)	N/A

∗ This describes the operative techniques required for treatment of included upper-extremity injuries. A *P* value less than 0.05 is considered statistically significant.

### Hospital usage

Patients postincrease had longer hospital LOS (13.54 ± 30.21 days vs 3.81 ± 3.15 days preincrease, *P* = 0.003) as well as longer ICU LOS (6.36 ± 6.65 days vs 2.00 ± 0 days preincrease, *P* = 0.001, Table 4). Postincrease upper-extremity trauma patients required more operative events (2.15 ± 2.10 vs 1.44 ± 0.73 preincrease, *P* = 0.050). Although the two groups had similar total operative time for all injuries, the postincrease group had increased operative times specifically for upper-extremity injuries (181.92 ± 130.79 minutes vs 104.29 ± 61.69 minutes preincrease, p=0.020). The average cost for each patient’s hospital stay adjusted for inflation also quadrupled after the increase in wall height ($397,631.95 ± $1,057,574.31 vs $98,978.32 ± $84,168.50 preincrease, *P* = 0.008). The total cost, adjusted for inflation to 2015 US dollars, was $1,583,653.15 preincrease and $37,377,403.18 postincrease, representing a 23-fold increase in health care expenditures in caring for these patients.

**Table 4:** Hospital Usage This describes the rate of use of hospital and operating room resources for care of patients with border wall injuries. A *P* value less than 0.05 is considered statistically significant

	2015–2018 (n = 16) Avg ± SD or num (percent)	2019–2022 (n = 94) Avg ± SD or num (percent)	*P* Value
Hospital LOS (days)	3.81 ± 3.15	13.54 ± 30.21	0.003
ICU LOS (days)	N = 3 2.00 ± 0.00	N = 25 6.36 ± 6.65	0.001

Operating room usage			
OR needed	9 (56%)	71 (76%)	0.132
Number of OR trips	1.44 ± 0.73	2.15 ± 2.10	0.050
Total OR time (minutes)	271.33 ± 194.33	341.51 ± 324.49	0.367
OR time for upper-extremity injuries (minutes)	N=7 104.29 ± 61.69	N=49 181.92 ± 130.79	0.020

Hospital costs			
Cost of hospital stay per patient, adjusted for inflation (dollars)	98,978 ± 84,169	397,632 ± 1,057,574	0.008
Cost of hospital stay, total	$1,583,653	$37,377,403	
Follow-up	1 (6%)	18 (19%)	0.297

SD, standard deviation; LOS, length of stay, ICU, intensive care unit; OR, operating room.

## Discussion

Construction on the United States-Mexico border wall was completed in 2019, resulting in a near-doubling of over 400 miles of barrier height meant to deter unauthorized crossings. Instead, the number of patients presenting to our Level I trauma center with border fall-related upper-extremity injury increased by 488% following the increase in wall height. Although upper-extremity fracture characteristics and the rates of dislocations, lacerations, and arthrotomies were not significantly different, there was a significant worsening in overall ISS with increased operative times and number of surgeries. These findings are in line with prior research that has shown greater levels of morbidity and mortality with increasing heights of fall-from-height trauma. A previous retrospective review of adult fall-from-height patients identified 25 feet as a transition point above which the likelihood of severe injury or death is significantly increased. Falls from >25 feet were also predictive of higher ISS and longer LOS in their cohort, a telling finding given the increase in border wall height to 30 feet during the course of our study.^21^ Another retrospective study found fall patients with fall height >5 meters (approximately 16 feet) and their body position on impact were predominant factors associated with more severe injury.^22^ Various mechanisms explaining the increased injury severity at greater heights have been proposed, including changes in landing pattern as well as the increased velocity, force, and secondary impact trauma after the initial landing.^17^^,^^23^ Higher ISS has been associated with worse long-term outcomes, including limb amputation, among patients with upper-extremity trauma.^24^

We also observed an increase in hospital resources used for injury management after the border wall height increase. Hospital LOS, ICU LOS, and number and length of operating room procedures all increased following the increase in barrier height. A resultant 23-fold increase in hospital charges was observed, translating to $36 million more in charges after the increase in border wall height. The actual cost of this cohort’s border fall injuries is likely much greater than the direct health care costs described here. Patients with upper-extremity injury are particularly limited in their ability to participate in the workforce and incur significant indirect costs because of decreased productivity.

Poor access to rehabilitation and resulting functional limitations may put this cohort at increased risk for financial hardship. Injured migrants who present to the trauma bay via ambulance or border patrol are able to receive emergency medical services regardless of immigration status or financial means. However, this level of access does not apply to rehabilitation programs, follow-up visits, or health care maintenance, all of which can directly impact the healing process and long-term outcomes following injury. Even in California, where many immigrants may be eligible for health coverage through the state’s Medi-Cal program, many may be unaware of this or hesitant to apply.^25^ Furthermore, those migrants who are ultimately detained or transported outside of San Diego postdischarge will face significant logistical barriers to maintaining their care. The severity of this cohort’s injuries only increases the importance of postdischarge rehabilitation and follow-up. Given the poor access to follow-up care observed in this study (only 6% of patients preincrease and 19% postincrease had a documented postdischarge visit), it is unknown but unlikely that patients are attaining optimal function.

There are several limitations to our study. First, the analysis includes patients admitted to only one of two trauma centers that received patients with border-related injuries in the county, although this is the primary trauma center serving this patient population. Second, hospital charges were used as a proxy for costs of care, though these values may differ. Third, the generalizability of this work is limited to land border crossings in San Diego and Imperial counties. The 450 miles of barrier that underwent construction during this study period account for just 23% of the total 1,954 miles of United States-Mexico border. Other sites may differ significantly in the types of barriers used, the rates and methods of attempted border crossings, and the access to health care for injured migrants. Furthermore, the height from which patients included in this study fell cannot be known and therefore any differences in the pre- and postincrease groups cannot be directly attributed to an increase in fall height. Lastly, the impact of changes in immigration or public health policy that occurred around the time of border wall expansion were not included.

This retrospective review adds to the growing body of literature exploring the public health dimension of immigration along the southern border and describes an increase in injury severity and surgical resource utilization among patients who sustained upper-extremity injury falling from a heightened border wall. Not only do the traumatic injuries reported here have significant impacts on patients’ abilities to function in their personal and professional lives, but the increased resource demand of treating these injuries places additional stress on an already strained health care system. The costs, supplies, and personnel required to treat border-related injuries should be considered by policymakers. Further investigation is warranted to determine how best to support migrants with recovery and hand therapy following upper-extremity injury. The poor access to rehabilitation services demonstrated here will likely have serious consequences for individual function as well as socioeconomic trajectory, although it is difficult to quantify these outcomes because of a limited ability to longitudinally follow this cohort.

Despite the increase in border wall height, the rate of border crossings along the southern border of the United States is projected to continue to increase through 2023 and 2024. The strain that this influx of migrants places on the state and local health care system and economy is only intensified when severe injuries are sustained in the crossing. Health care providers, especially in areas that see a high volume of border wall-related injuries, should continue to investigate the trends, clinical needs, and outcomes of this population in order to improve patient care.

## Conflicts of Interest

No benefits in any form have been received or will be received related directly to this article.
